# The positive impact of COVID-19 on critical care: from unprecedented challenges to transformative changes, from the perspective of young intensivists

**DOI:** 10.1186/s13613-023-01118-9

**Published:** 2023-04-11

**Authors:** Bertrand Hermann, Sarah Benghanem, Youenn Jouan, Antoine Lafarge, Alexandra Beurton, the ICU French FOXES (Federation Of eXtremely Enthusiastic Scientists) Study Group

**Affiliations:** 1 Service de Médecine Intensive - Réanimation, Hôpital Européen Georges Pompidou (HEGP), Groupe hospitalo-universitaire Assistance Publique - Hôpitaux de Paris, Centre - Université Paris Cité (GHU AP-HP Centre - Université Paris Cité), Paris, France; 2 Faculté de Médecine, Université Paris Cité, Paris, France; 3 INSERM U1266, Institut de Psychiatrie et Neurosciences de Paris (IPNP), Paris, France; 4 Service de Médecine Intensive - Réanimation, Hôpital Cochin, Groupe hospitalo-universitaire Assistance Publique - Hôpitaux de Paris, Centre - Université Paris Cité (GHU AP-HP Centre - Université Paris Cité), Paris, France; 5 Service de Médecine Intensive - Réanimation, CHRU Tours, Tours, France; 6 Service de Réanimation Chirurgicale Cardiovasculaire & Chirurgie Cardiaque, CHRU Tours, Tours, France; 7 INSERM U1100 Centre d’Etudes des Pathologies Respiratoires, Faculté de Médecine de Tours, Tours, France; 8 Service de Médecine Intensive - Réanimation, Hôpital Saint Louis, Groupe hospitalo-universitaire Assistance Publique - Hôpitaux de Paris, Nord - Université Paris Cité (AP-HP Nord - Université Paris Cité), Paris, France; 9 Service de Médecine Intensive - Réanimation, Hôpital Tenon, Groupe hospitalo-universitaire Assistance Publique - Hôpitaux de Paris, Sorbonne Université (GHU AP-HP Sorbonne Université), Paris, France; 10 Service de Médecine Intensive - Réanimation, Hôpital Pitié Salpêtrière, Groupe hospitalo-universitaire Assistance Publique - Hôpitaux de Paris, Sorbonne Université, Paris, France; 11 UMRS 1158 Neurophysiologie Respiratoire Expérimentale et Clinique, Sorbonne Université, Paris, France

**Keywords:** Coronavirus disease 2019, Pandemic, ICU, Intensivists, Well-being

## Abstract

Over the past 2 years, SARS-CoV-2 infection has resulted in numerous hospitalizations and deaths worldwide. As young intensivists, we have been at the forefront of the fight against the COVID-19 pandemic and it has been an intense learning experience affecting all aspects of our specialty. Critical care was put forward as a priority and managed to adapt to the influx of patients and the growing demand for beds, financial and material resources, thereby highlighting its flexibility and central role in the healthcare system. Intensivists assumed an essential and unprecedented role in public life, which was important when claiming for indispensable material and human investments. Physicians and researchers around the world worked hand-in-hand to advance research and better manage this disease by integrating a rapidly growing body of evidence into guidelines. Our daily ethical practices and communication with families were challenged by the massive influx of patients and restricted visitation policies, forcing us to improve our collaboration with other specialties and innovate with new communication channels. However, the picture was not all bright, and some of these achievements are already fading over time despite the ongoing pandemic and hospital crisis. In addition, the pandemic has demonstrated the need to improve the working conditions and well-being of critical care workers to cope with the current shortage of human resources. Despite the gloomy atmosphere, we remain optimistic. In this ten-key points review, we outline our vision on how to capitalize on the lasting impact of the pandemic to face future challenges and foster transformative changes of critical care for the better.

## Background

Over the past 2 years, more than six million deaths due to Coronavirus Disease 2019 (COVID-19) have been recorded worldwide [1] and this death toll may even be significantly underestimated [2]. The pandemic has led to an unprecedented increase in the number of patients admitted to intensive care units (ICU) [3, 4]. As young critical care caregivers in the early stages of their professional careers, we have been and remain at the forefront of the management of this enduring health crisis. While still in the throes of the current pandemic, it has already disrupted our newly acquired vision of critical care [5]. Since this disruption is likely to be systemic, the pandemic will have a lasting impact on critical care and it is important to introspect without further ado, in order to actively build up the framework for tomorrow's critical care.

**Fig. 1:**
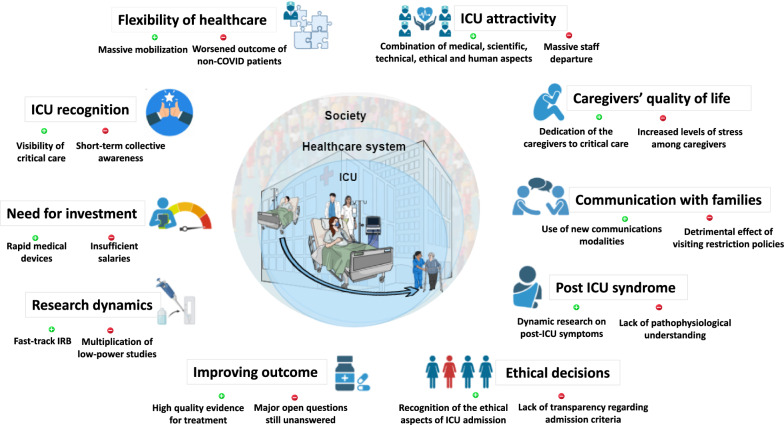
The positive impact of COVID-19 on critical care

In this review, we examine how critical care has coped with some of the challenges posed by this pandemic (Fig. 1 and Table 1) in ten key points, and we make proposals for how we should capitalize on this experience to foster the transformative changes we hope for to provide better critical care, for caregivers and patients alike.

## Main text

### Central role and adaptability of intensive care within the healthcare system

To face this unprecedented influx of patients, the whole healthcare system was forced to adapt, from hospital to ambulatory care. Indeed, according to a 2018 international annual report, nearly half (43%) of 182 countries were not prepared to prevent, detect and control the outbreak of a new infectious disease [6]. The chaos of the first few weeks of the pandemic revealed that actually no countries were operationally equipped to deal with an outbreak of such magnitude. Given the potential severity of the disease, intensive care had a central role to deal with this pandemic and was on the frontline of a massive reorganization [7, 8]. All across the world, different strategies were set up to lower the pressure on overwhelmed ICUs [9–11], from the creation of temporary ICU beds through repurposing of non-critical care beds [12–15] and upgrading intermediate care beds to admit severe intubated patients, to inter-hospital transfer of critically ill patients, even across borders (Table 2). The latter strategy appears to be both effective and safe [16–18]. However, it may have increased the emotional burden on families and its cost-effectiveness is more than questionable in comparison to increasing ICU capacities. Moreover, this strategy may not be feasible in a pandemic in which all regions/countries are similarly affected.

**Table 1:** Impact and potential legacy of the COVID-19 pandemic on critical care

Domain	Positive	Drawbacks	Proposals
Flexibility of critical care	• Healthcare workers’ mobilization • Healthcare system reorganization • Pivotal role of intensive care	• Suboptimal management of COVID-19 patients • Worsened outcome in non-COVID patients	• Predetermined crisis response plan • Coordinated medical leadership • Preplanned activity continuation plan • Expandable and monitored ICU bed capacities
Need for investment	• Massive investments • Rapid responses from politicians • Resource reallocation by industry	• Shortages of human resources and medical devices • Deficient public–private partnership • Lack of international solidarity	• Reinforced public–private partnership • Improved monitoring of equipment stocks • Wage revaluation • Increased ICU caregiver staffing resources
Visibility of critical care	• Social recognition • Intensivists’ role in public health • Direct communication through social networks and media	• Caregivers’ harassment • Scientific controversy • Unclear boundaries between science and politics	• Media communication training • Medical and scientific education for the general population • Designated spokespersons from professional union organizations • Institutional boards including paramedical staff
Research dynamic	• Massive scientific production • Early patient enrollment in large RCTs • Fast-track IRB and peer-review process	• Lower scientific standards • Study duplication • Negative impact on non-COVID scientific production	• Large scale intensive care registries • Improvement of clinical research coordination • Harmonized and simplified process of ethical approval • Improved transparence (open-reviews, data and analysis sharing…)
Improving outcome	• Beneficial impact of corticosteroids • Targeted interventions • Enhanced non-specific supportive care strategies	• Few treatments for critically ill patients • Failure of bio-plausible treatments • Use of non-validated treatments	• Evaluation of non-specific supportive interventions in non-COVID-19 ARDS • Avoid treatment use before large-scale and methodologically rigorous trials • Personalized and precision medicine integrating disease time-course, clinical phenotypes, omics tools, biomarkers
Ethical decisions	• Early concerns about admission strategies • Recognition of the ethical aspects of ICU admission • Understanding of the complexity of ethical decisions	• Inadequacy between massive influx and limited ICU bed capacities • Use of single criterion triage (notably age) • Lack of transparency in admission criteria	• Dedicated triage team • Anticipated decisions with family and referring team • Stand-by resuscitation with rapid and regular reassessment of healthcare goals • Prognostic score development based on patient-centered outcome
Post-intensive care syndrome	• Increased recognition of post-intensive care syndrome • Research dynamics on post-ICU symptoms	• Lack of systematic post-ICU follow-up • Insufficient information on long-term outcomes • Lack of pathophysiological data	• Focused research on PICS pathophysiology • Post-ICU multidisciplinary follow-up • Expanded capacities of rehabilitation facilities
Communication with families	• New multimedia communication tools • Early and gradual lifting of visit restrictions • Renewed demonstration of the importance of in-person visits	• Increased PTSD and depression incidences in patients and families • Increased burn-out incidence in ICU caregivers	• Open-door ICU • Additive channels of communication • Dedicated focusing research • Secured digital tool development
Caregivers’ quality of life	• Awareness of healthcare workers' well-being • Impressive resilience by caregivers	• Altered working conditions • Increased burn-out incidence • Unsuitable interventions	• Access to well-being programs • Recognition of the difficult working conditions • Increased staffing resources • End of 24h rounds
ICU attractivity	• Unprecedented media coverage • Social recognition • Caregiver solidarity	• Decreased staffing resources • Aggravation of the hospital crisis	• Implemented diploma and training programs dedicated to caregivers • Wage revaluation • Respectful and caring working environment

*ICU* intensive care unit, *IRB* institutional review board, *RCT* randomized controlled trial

**Table 2:** Accommodating ICU bed capacities to face a rapid surge of patients

	Available means	Challenges and limiting factors
LOCAL LEVEL		
Coordination	• Identify and reduce non-essential activities	• Risk of delays in management and worsened outcome of non-epidemic patients
	• Preplanned crisis and activity continuation plan	• Intrahospital cooperation with medical leadership • Allocation of staffing resources according to their qualifications
Increasing ICU capacity	• Repurposing of other critical beds	• Continuation of non-epidemic activity
	• Repurposing of non-ICU beds - operating rooms - emergency departments - regular wards	• Need for trained staffing resources • Need for medical devices and equipments • Monitor patients’ safety
	• Creation of ephemeral beds in new locations	
REGIONAL & NATIONAL LEVEL		
Dispatch patients to avoid overwhelmed hospitals	• Real-time follow-up of available beds	• Uniformization of applications among centers • Manpower to fill registries in real-time
	• Inter-hospital transfer - intra-regional - inter-regional - international	• Safe transportation of critically ill patients • Human resources

Another lever of this reorganization was the redistribution of tasks and reallocation of resources from less essential activities, which was not without detrimental consequences. First, the suboptimal management of patients in structures not initially designed to host critically ill patients [19, 20] had a well demonstrated negative impact on the outcomes [21, 22]. And second, the reorganization of care to almost ‘‘100% COVID-19’’ was done at the expense of non-COVID-19 patients [23, 24], in particular patients with chronic diseases, such as cancer patients awaiting chemotherapy or surgery [25–27] (Table 2). Lastly, a reduction of Emergency Department visits was observed with an increased out-of-hospital mortality [28, 29].

While the COVID-19 pandemic is still active today, and in order to deal with future outbreaks, terrorist attacks [30–32], or environmental threats such as heat waves [33, 34], and in addition to investments in equipment, funding should support flexible ICU designs and expandable ICU bed capacity. An efficient approach to public–private partnership combined with a centralized health care system would improve the redistribution of medical devices and human resources in response to fluctuations in demand [5, 48] (Table 3). Critical care flexibility should be anticipated via predefined crisis protocols not only on a national and regional scale, but also on a local scale. Such protocols should identify a coordination team in advance, composed of both caregivers and administrative staff. It should also include stepwise measures to increase ICU capacity on the scale of each institution while providing continuity of essential tasks when coping with the influx of patients [35, 36]. In addition, critical care should be coordinated region- and nation-wide with the development of online registries allowing for real-time tracking of available ICU beds through an intuitive web application, such as COORD-REA® or the *Repertoire Opérationnel des Ressources de l’offre de santé* [37–40] in France, to facilitate the dispatching of patients in private or public institutions and prevent center overcrowding (Table 3).

**Table 3:** Top ten measures to improve critical care 449 over the next 10 years 450

Top ten measures to improve critical care over the next ten years	
Crisis protocol and decision-making process with a coordination team must be anticipated, drafted at local, regional, national levels and must be communicated to all healthworkers to avoid stress and confusion	
Efficient public–private partnership combined with a centralized health care system approach should improve the redistribution of medical devices and human resources	
Development of international collaborations with harmonized institutional research design	
Patient-centered collective shared-decision process with an independent expert must be discussed in regular multidisciplinary meetings, regularly updated and written in the patient’s medical record	
Promote the culture of advance directives through improved communication with national communication campaigns	
Improve healthworkers’ ethics education	
Improve post-ICU care, including respiratory, neurologic and psychological rehabilitation with a multidisciplinary approach	
Develop communication with the family by improving digital tools	
Increasing staff resources must be a priority to prevent work-related stress and remain attractive	
Create a positive and motivating daily environment (well-being program, flexible schedules, reduce administrative tasks)	

As a result, critical care has had a central role in patient management and within the hospital health care system resulting in unprecedented visibility for our specialty.

### Unprecedented visibility and recognition of intensive care

During the COVID-19 pandemic, and for the first time [41, 42], major decisions were taken by public health institutions in partnership with intensivists, including containment measures, vaccination strategies, welfare policies, healthcare system management and coordination to prevent ICU overflowing [43] (Table 1). We should be proud of the intensivists’ role in public health and their shared responsibility with governments [44].

Alongside policy makers, ICU caregivers have also gained recognition in the media through daily televised interventions, newspaper editorials [45–47] and social media involvement [48]. Here again, these interventions have positively contributed to the notoriety of intensive care. Yet, they have sometimes led to premature claims and misunderstandings that we should be careful to avoid in the future. To this end, we believe we should resist the siren’s call of short-term commentary when no scientific evidence exists. We should also prioritize long format and pedagogic interventions allowing to present controversies and doubt as one of the cornerstones of scientific reasoning and legitimate debate rather than a mere expression of division and incompetence that would ultimately lead to distrust [49]. Following this line of thought, we should also encourage every initiative to spread scientific culture that is too often lacking, including among the policy makers [50], through interventions in educational programs or in high-quality popular science channels that are flourishing on the web. Social media could rightly serve this purpose and we should definitely seize this opportunity to be in direct contact with the public. However, this will require adequate training to avoid some of the pitfalls of these new communication channels and protect ourselves against the fierceness of social networks [51]. Leading this media and digital campaign should be a priority of our academic organizations and professional unions to increase the visibility of all critical care professions.

As time passes by, we fear that the social legitimacy obtained during the first wave ultimately turns out to be more emotional than a genuine collective awareness of our importance in healthcare. Even if this was not the case, we may wonder how long this memory will last as indifference seems to be growing while people are still dying in the ICU during an umpteenth wave. Now that we have a foot in the door, we should all pursue the collective effort to nurture this unprecedented role of intensivists in public life.

### Outstanding research dynamics

Thanks to political decisions and the mobilization of medical and scientific communities, the extraordinary dynamics of clinical, fundamental and translational research has been one of the genuine breakthroughs in the fight against COVID-19 [52] (Table 1). Since the early 2020s, the human investment in research from all specialties has led to a better understanding of the virus, its pathophysiology, and its impact on dysfunction organ allowing for better patient management in record time. Critical care research has been particularly efficient, accounting for more than 10% of global COVID-19-related publications [53, 54]. The surge in research has been supported by combined efforts of critical care professionals, fast-track institutional review processes and ethics committee approval [45]. Early large-scale studies including critically ill COVID-19 patients have impacted COVID-19 therapeutic strategies [7, 55–58]. The development of international collaborations, adaptive and pragmatic designs, and the use of platform trials have enabled practice-changing trials such as the Randomised Evaluation of COVID-19 Therapy (RECOVERY) which enrolled over 10,000 patients in 176 hospitals within three months [59]. In addition, the World Health Organization and international intensive care societies have been extremely efficient in incorporating this growing body of evidence into guidelines, for the benefit of the whole community, both researchers and civil society [60].

Yet, this era was also marked by a massive scientific production, with sometimes low standards and several design pitfalls that generally lead to inconclusive or futile results, e.g., insufficient power to prove working hypotheses, flexible endpoints subject to assessment bias in open-label studies, lack of appropriate comparators, non-randomized allocation of treatments, duplication or fragmentation of data, and retrospective analyses of observational data. This is how the use of treatments such as hydroxychloroquine was promoted, whereas it has ultimately been proved to be detrimental in high-quality clinical trials [61] (Table 1). This should be a strong reminder that all treatments should be tested in methodologically rigorous trials prior to their implementation in clinical practice, even in case of strong biological plausibility and presumed safety [62]. The scientific community, as well as civil society and politicians, have become aware of this methodological disparity and the hazardous conclusions, leading to distrust of the medical profession, which we experienced during the vaccination campaigns. Methodological education in response to the quality of COVID-19 publications has been published [63]. Rushing is not good for scientific rigor and in this sense, indeed, fast-track procedures have been abandoned.

Lastly, research on COVID-19 has affected the production of non-COVID-19 research. This phenomenon, criticized by many key opinion leaders, may be due to a redirection of funding allocations, shifts in editorial strategies and limited access to patients during containment periods [64, 65].

Intensive care clinical research will surely benefit from these advances. National intensive care registries would become systematic, promoting large-scale adaptive studies worldwide [63, 66, 67]. The rigorousness of methodological requirements combined with improved clinical research coordination would curb the risk of duplication and underpowered studies while providing a faster, simplified process of ethics approval. International research collaborations would benefit from harmonized institutional review board procedures [68] (Table 3). A transparent editorial and an open-access review process would contribute to effective knowledge sharing which would be incorporated into real-time international guidelines.

### Global improvement of critically ill patient outcome

The abundant research has led to conclusive evidence to improve patient outcome in an extremely short period of time [69]. In patients requiring ICU admission, dexamethasone [59, 70], IL-6 receptor antagonists [71] and neutralizing antibodies for Delta and Omicron variants [72–74] have shown a significant beneficial impact. The heterogeneity in critically ill COVID-19 patients’ response to corticosteroids and the disappointing results of other therapeutic interventions underscore the importance of the timing of treatment onset with respect to the disease course of inflammatory response and lung injury [75].

Another improvement during the pandemic was the shift in therapeutic strategy from a one-size-fits-all approach to more targeted interventions in subpopulations such as patients with high prevalence of underlying immune effect [76], B-cell lymphoid malignancies [77] or seronegative patients [74] illustrating the necessary evolution of intensive care towards personalized and precision medicine [78]. Post-COVID-19 critical care strategy will integrate disease time-course, clinical phenotypes, omics tools and new biomarkers in order to rapidly detect treatment responders and avoid immunomodulatory side effects in others.

In parallel, outcomes of critically ill patients have mostly been improved by refinement of non-specific supportive care strategies such as high-flow nasal cannula oxygen and non-invasive ventilation [79–83], awake prone-positioning [84, 85], all of which were not as widely used for the treatment of acute respiratory failure due to non-COVID-19 pneumonia. Non-COVID-19 patients will benefit from these therapeutic breakthroughs, as this progress in non-specific supportive interventions is most likely to be broadly applicable to patients with non-COVID-19 acute respiratory distress syndrome (ARDS).

### Innovative communication with patients and families

The first waves of the pandemic, marked by lockdowns, social distancing measures, visit restrictions and even bans, undermined “patient- and family-centered care”. The restrictions led to extreme seclusion situations for ICU patients, although it is proven that a flexible family visitation policy is associated with a better patient experience of the ICU stay and a potential reduction of delirium and anxiety symptoms [86–88]. These restrictions also negatively impacted patients’ relatives due to limited access to medical teams and subsequent limited information [89, 90]. Here again, healthcare professionals showed unprecedented adaptability, making use of new multimedia communication tools with video-calls and virtual visits [91] as well as writing and drawing in daily diaries [87] (Tables 1, 3). Moreover, hindsight in the aftermath of the pandemic and the effectiveness of vaccination campaigns and social distancing measures have allowed for a gradual lifting of visiting restrictions for relatives to complete reopening, notably for dying patients. This should remind us that face-to-face communication remains the gold standard for all.

The negative experience of visiting policy restrictions during the pandemic period clearly supports the adoption of a flexible opening policy, ideally not less than 12 h per day. Moreover, the production of structured effective communication guidelines [87, 92, 93] associated with new possibilities offered by multimedia tools to maintain connections between patients and relatives, and between families and medical staff, will undoubtedly change the future of critical care communication strategies, always prioritizing patient and family comfort, well-being and quality of life [94].

### Facing ethical dilemmas: from individual to collective choices

Early on, the pandemic highlighted some key ethical issues regarding life support withdrawal decisions, quality of end-of-life support and above all admission strategies (i.e., triage) [95–97] (Table 1). These issues were particularly related to the extreme strain on ICU beds, given that delayed ICU admission due to a full unit is associated with increased mortality [98].

Good practice regarding patient admission recommends a patient-centered collective shared-decision process involving the referring physicians and taking into account the patient’s premorbid conditions, frailty and anticipated prognosis of the acute illness as well as their wishes concerning their quality of life and the degree of disability they are willing to accept [7, 99] (Table 3). The overall goal of this multifactorial approach is to propose a tailored, personalized "treatment plan", which can be re-evaluated during the ICU stay according to the patient’s progression, thereby allowing for transparent communication with the patient and his family regarding the goals of care.

However, it should be acknowledged that these good practices have not always been followed with disparities between centers and countries. It has been suggested that the high between-center heterogeneity of patient trajectories and outcomes may be at least partially due to a wide disparity in triage criteria [100], including age [96, 101]. It should be pointed out that the personalized benefit/risk balance of triage decisions in an unprecedented situation with a lack of evidence regarding disease progression, prognostic factors, and potential sequelae is a difficult matter, and that simple severity scores developed outside COVID have been shown to be inaccurate [102]. Although it is desirable to develop more accurate and earlier prognostic tools based on patient-reported outcome measures, it is misleading to believe that a single criterion and/or score could ever supplant the complex decision-making process guiding ICU admission. As there is no universal consensus on some of the basic principles that should prevail [103], we should rather acknowledge this complexity and promote distributive justice, postulating fairness and equity in the allocation of resources, accounting for potential socioeconomic and demographic inequities [104, 105], especially in case of ICU bed shortages [106, 107]. It has been proposed that critical care teams responsible for patient care should be relieved of the responsibility for admission or non-admission decisions. The decisions must be informed by objective elements that can change over time, taking into account the opinion of the patient (or, failing that, the family) throughout the course of treatment. This work would thus be performed by a dedicated triage team [108, 109]. The advantage of this approach is that it avoids the emotional impact of choosing whether or not to admit a patient to an ICU [110]. However, the composition of these teams must be specified to avoid hurting and/or guilt for the health care team [107, 111].

All these debates, which were not restricted to caregivers, revealed the complexity of emergency ethical decisions to the general population and political stakeholders, and they should ultimately be beneficial in improving our daily ethical practices beyond the COVID-19 pandemic. But most importantly, the general population must be encouraged to participate in the discussion, firstly through national communication campaigns, and also through anticipated decisions with the family and the referring team (Table 3). Indeed, each patient followed for a chronic disease at risk of worsening and each hospitalized inpatient should be informed and encouraged to express his/her wishes regarding the goals of care via advance directives (Table 3). Patients’ wishes should be written in the patient’s medical record, accessible to all practitioners and communicated to all physicians in charge of the patient. The shared-decision process should be implemented during regular multidisciplinary meetings and updated throughout the time course of the disease. Moreover, in each structure, a dedicated team of independent experts from various fields as well as non-experts from the civil society should be available as recourse for difficult cases. Lastly, ethics education should be reinforced by specialized courses in continuing medical education, by participation in ethics boards of critical care societies, and in debate sessions during national and international symposiums.

### Post-intensive care syndrome awareness

Prior to the pandemic, a growing body of evidence had already been accumulated regarding potential persistent disabilities in ICU survivors, notably after sepsis and ARDS [112–118], pooled together under the concept of ‘Post-Intensive Care Syndrome’ (PICS) [119–121] (Table 1). These sequelae range from physical disabilities such as gait disorders and fatigue due to ICU-acquired weakness and/or persistent organ dysfunction such as kidney, cardiac or respiratory failure, to psychiatric and cognitive disorders, all impairing the quality of life of ICU survivors. Their pathophysiology remains poorly understood, but intensity of the initial episode with persistent inflammatory and metabolic alterations are thought to be important mechanisms [122]. PICS is observed in approximately half of ICU survivors [123], and prior to the pandemic, considerable efforts had been made to improve PICS recognition and management [124]. However, despite multi-organ involvement of PICS and a myriad of negative consequences for patients, increasing PICS awareness was mostly limited to critical care and rehabilitation communities. With thousands of ICU survivors discharged from hospital after severe COVID across countries after the first waves, this question became relevant to all [125].

Thus, recent reports have shown that incidence of PICS following severe COVID-19 is particularly high [126–128], with clinical features similar to non-COVID acute respiratory failure requiring invasive mechanical ventilation [129]. Patients with COVID-19 ARDS have been shown to require more sedation (propofol and benzodiazepine) than non-COVID-19 patients to achieve the same median levels of sedation [130, 131]. In this context, early mobilization of mechanically ventilated patients, daily discontinuation and/or nurse-protocolized targeted sedation, management of physical and psychological discomfort and avoidance of prodelirious drugs are crucial points in ICU patient rehabilitation [132]. Despite growing recognition, PICS management is likely to be insufficient after ICU discharge due to the lack of specific structures and data regarding how post-ICU follow-up should be organized [119, 124]. Though data from the pandemic have not yielded definitive answers for these critical questions, increased global PICS awareness will help us to communicate not only with patients and their relatives regarding PICS, but also with other care providers that might be involved in the management. At the end, the high prevalence and poor functional prognosis associated with COVID-19 highlight the urgent need for reorganization of post-ICU care, including respiratory and neurological rehabilitation with a multidisciplinary approach involving intensivists, rehabilitation physicians, physiotherapists, psychologists, organ subspecialists and general practitioners (Table 3).

### Massive material and human investments in intensive care

The fight against COVID-19 was marked at the onset by an unparalleled mobilization—both in terms of scale and speed—of human, material and financial resources (Table 1). Countries spent trillions of dollars to support the economic cost of containment measures but also to purchase personal protective equipment, ICU devices such as ventilators, consumables and pharmaceuticals [133, 134]. Facing major logistical constraints (shortages of medical devices, personal protective equipment and drugs, inadequate gas supply) and human challenges [135, 136], the extraordinary mobilization and joint efforts of medical, paramedical and administrative staff allowed to cope with the massive influx of patients. This mobilization helped to support enhanced ICU bed capacity while trying to maintain safe nurse/patient ratios and intensivist/patients ratios [137–139]. The youngest largely contributed to this effort with great flexibility and adaptability. Indeed, many trainees engaged in a research year interrupted their doctorate or master's degree to help at the bedside and students from nursing schools were sent into the field in a great participatory impulse.

Despite these efforts, many ICU departments faced major shortages of human resources and medical devices, jeopardizing both patients’ and healthcare workers’ safety [140]. Moreover, the response of healthcare systems to the COVID-19 pandemic was hampered by a lack of public policy coordination both at national and international levels, deficient cooperation between governments and industry [5, 141] and the lack of international solidarity. It is now our responsibility to capitalize on this acknowledgment so as to remind the policy makers and civil society how important it is to build resilient and effective critical care that will need to outlast the current pandemic [142]. The public financial concessions achieved so far will not be enough.

Efforts should also be made to align research funding in critical care to the financial burden of critical illnesses [143]. But above all, if we want to be able to provide high-quality care in the future, focus should be on the investment in human resources, to increase the safety of patients, the well-being of caregivers and the overall attractiveness of critical care.

### Caring for caregivers is a health priority

Awareness of the paramount importance of healthcare workers’ well-being has taken on a very new scope with this health crisis [144, 145] (Table 1). Working conditions during the pandemic were negatively impacted by many factors, notably a heavier workload (high number of patients, organizational changes with an increase in ICU beds usually not matched by an adequate increase in staffing resources) and a high emotional burden (high, persistent levels of stress due to the uncertainty about the evolution of the pandemic, shortage of personal protective equipment, difficult ethical triage decisions, fear of being sick and of transmitting the virus to relatives) [144–150]. The 2020 National Physician Burnout and Suicide Report showed a 44% rate of burnout among ICU physicians [151]. The COVID-19 pandemic has increased this rate not only due to dying patients [152] but also to additional physical and psychological demands with a poor recognition of their work [153, 154]. We are at a watershed moment for caregivers and proffering “resilience” as the solution to the burn-out crisis is no longer acceptable [155].

To improve healthcare professionals’ ICU experience, policy makers should provide a more motivating, positive work environment to foster emotional well-being and empathy [156, 157] through multiscale interventions [158]. Notably, fostering communication among the team and with external consultants (dedicated training in communication and conflict resolution, multidisciplinary rounds [159]) and giving access to well-being programs [148, 160, 161] should be promoted to mitigate burn-out symptoms. But, more importantly, increasing staffing resources appears to be a prerequisite to prevent work-related stress [148]. It is indeed a priority to train and recruit physicians, nurses, nursing auxiliaries, psychologists, physical therapists and secretaries to allow for part-time work with flexible schedules [162], to reduce the burden of administrative tasks, to foster teamwork and the strengthened nurse–physician pairing, and to encourage communication and open dialogue concerning mental health issues. Emphasis should now be put on matching healthcare workers' cognitive assessment of the perceived demands with their perceived capability, skills and resources to deal with those demands [144, 146, 148].

The involvement of all stakeholders will be needed to guarantee a better understanding of caregivers’ expectations, which will be the main concern for the next generation of ICU healthcare professionals and an absolute necessity for critical care to remain attractive (Table 3). It is definitely time to care for caregivers.

### The challenge of intensive care attractiveness

As previously discussed, the omnipresence of the pandemic in everyday life with its associated coverage in the media shed light on our specialty, hitherto unknown to the public. The community became aware not only of our highly technical environment and the very specific skills it requires, but also of our genuine dedication to patients and their families, especially the dedication of the youngest physicians, who have little media exposure but were at the bedside. Society became aware of our resilience [136] (Table 1). Yet, after a brief period of recognition and applause by the general population, and government promises of improved working conditions, we must acknowledge that ICU attractiveness has not improved and may even have deteriorated. The pandemic has exposed and aggravated the long-lasting hospital crisis, hence accelerating the massive departure of physicians, nurses and nurse assistants from the ICU and from public hospitals to private structures, or even engaging in professional retraining due to perceived loss of meaning [163, 164].

Nevertheless, we remain hopeful that the pandemic and this spotlight on our specialty will help to attract young healthcare workers interested in complex pathophysiological concepts and in patients with multiple organ failure, who wish to provide state-of-the-art multidisciplinary care combining advanced techniques while being constantly concerned by the ethics of care. The two main levers to achieve this are firstly a substantial increase of nurses and nurses assistants’ wages and secondly a better recognition of the specific skills of critical physicians, nurses and caregivers, opening opportunities for career development. This recognition will require the development of dedicated diploma (such as the recent advanced nurses practitioners status) and training programs for all caregivers [165, 166] (such as European Society of Intensive Care Medicine and Société de Réanimation de Langue Française training programs).

In the end, we believe that recognition of the specific challenges of intensive care medicine by society, political leaders and other medical specialties, will promote the attractiveness of critical care medicine [136]. It is time for our critical care community to take advantage of this publicity to nurture vocations and foster the emergence of young critical care leaders. We invite you to participate in on our optimism and join our group of motivated young intensivist (icufrenchfoxesstudygroup@gmail.com).

## Conclusion

The unprecedented COVID-19 pandemic has upset the convictions and beliefs of all: caregivers, politicians and society alike. From the initial diagnosis of severe SARS-CoV-2 infection to the management of organ failure in the ICU to hospital discharge, the intensive care medicine community assumed a major role in the care pathway of patients. This central place has highlighted three essential points: the difficulty for intensivists to decide whether or not to admit a patient in ICU despite regular use of pre-existing ethics committees, the now well described long-term consequences of critical care, and the importance of communication between caregivers, families and patients. Although these essential points had previously been acknowledged, the COVID-19 pandemic shed a new light on them. Critical care also took an essential place in the healthcare system thanks to its adaptability, the human investment, and the rapid research response to improve patient outcome. Pursuing these efforts, notably through the promotion of facilities and funding for further research will be warranted to maintain and strengthen these impressive achievements. Finally, intensive care became the focus of attention of the whole society. The caregivers’ resilience in such difficult and uncertain conditions were exposed in the media and social network, raising awareness of their dedication and priority to foster well-being at work in the midst of growing indifference as the pandemic pressed on.

Notwithstanding this gloomy atmosphere, we, as young critical care caregivers, are determined to maintain a positive and optimistic outlook on intensive care and we are convinced we can capitalize on these achievements to build better critical care. We realize that this hard-won new recognition should not be taken for granted and that it will have to be defended tooth and nail. This is thus a momentum for our specialty. Now is the time for us to actively engage to convert the lessons learnt from these unprecedented challenges into transformative changes. All colleagues are welcome to join in on our optimism.

## Data Availability

Not applicable.

## Abbreviations

ARDS: Acute respiratory distress syndrome
COVID-19: Coronavirus disease 2019
ICU: Intensive care unit
PICS: Post-intensive care syndrome
